# Time after Time: Temporal Variation in the Effects of Grass and Forb Species on Soil Bacterial and Fungal Communities

**DOI:** 10.1128/mBio.02635-19

**Published:** 2019-12-17

**Authors:** S. Emilia Hannula, Anna M. Kielak, Katja Steinauer, Martine Huberty, Renske Jongen, Jonathan R. De Long, Robin Heinen, T. Martijn Bezemer

**Affiliations:** a Department of Terrestrial Ecology, Netherlands Institute of Ecology, Wageningen, The Netherlands; b Institute of Biology, Section Plant Ecology and Phytochemistry, Leiden University, Leiden, The Netherlands; Stanford University; University of California, Berkeley

**Keywords:** environmental microbiology, fungi, plant-microbe interactions, plant-soil feedback, soil microbiology

## Abstract

Microorganisms are found everywhere and have critical roles in most ecosystems, but compared to plants and animals, little is known about their temporal dynamics. Here, we investigated the temporal stability of bacterial and fungal communities in the soil and how their temporal variation varies between grasses and forb species. We established 30 outdoor mesocosms consisting of six plant monocultures and followed microbial communities for an entire year in these soils. We demonstrate that bacterial communities vary greatly over time and that turnover plays an important role in shaping microbial communities. We further show that bacterial communities rapidly shift from one state to another and that this is related to changes in the relative contribution of certain taxa rather than to extinction. Fungal soil communities are more stable over time, and a large part of the variation can be explained by plant species and by whether they are grasses or forbs. Our findings show that the soil bacterial community is shaped by time, while plant group and plant species-specific effects drive soil fungal communities. This has important implications for plant-soil research and highlights that temporal dynamics of soil communities cannot be ignored in studies on plant-soil feedback and microbial community composition and function.

## Abstract (precis)

Our findings highlight how soil fungal and bacterial communities respond to time, season, and plant species identity. We found that succession shapes the soil bacterial community, while plant species and the type of plant species that grows in the soil drive the assembly of soil fungal communities. Future research on the effects of plants on soil microbes should take into consideration the relative roles of both time and plant growth on creating soil legacies that impact future plants growing in the soil. Understanding the temporal (in)stability of microbial communities in soils will be crucial for predicting soil microbial composition and functioning, especially as plant species compositions will shift with global climatic changes and land-use alterations. As fungal and bacterial communities respond to different environmental cues, our study also highlights that the selection of study organisms to answer specific ecological questions is not trivial and that the timing of sampling can greatly affect the conclusions made from these studies.

## INTRODUCTION

Ecological succession is defined as the process by which the structure of a biological community changes over time. Such temporal changes have been studied extensively for plant (1) and animal (2) communities to identify relationships between community stability and biodiversity (3) and predict community responses to disturbances (4). Though microorganisms are found everywhere and have critical roles in ecosystems, far less is known about the temporal dynamics of microbial communities, partly due to practical difficulties of measuring microbial species and because a clear definition of species is lacking and approaches vary greatly between studies (5, 6). However, high-throughput sequencing methods have greatly advanced our ability to measure microbial communities. This has led to an increase in longitudinal studies that examine variation and stability in microbial communities over time at different time scales (7–9).

Soils are especially rich in microorganisms, and given their importance in processes such as succession, temporal processes of microbes warrant future study (10). Microbiomes in many ecosystems are variable (11). For instance, infant gut microbiomes are highly variable and can change in a matter of hours to days. Soil ecosystems, on the other hand, are thought to be more stable. However, the appropriate time scales for soils vary greatly depending on the process assessed (8). After glacial retreat, it can take decades for pioneering microbial communities to reach a stable state, after which the community does not change much without further major disturbance (12, 13). However, in established soil microbial communities, the responses to root exudates at the individual plant level can also be rapid and are often visible within a few days to weeks (14, 15). Here, we are interested in monthly changes induced by different plant species and how this varies between grass and forb species.

In plant ecology, plants are frequently classified into grasses and forbs (these are called plant functional groups), because plant species that belong to the same “functional group” tend to have similar effects on ecosystem processes and respond similarly to environmental conditions (16). Forbs and grasses are known to have contrasting effects on soil microorganisms due to the exudation of different carbon compounds into the soil and their association with different soil organisms (17). This can also be related partly to different root traits in grasses and forbs. For example, grasses typically invest more resources into dense fibrous roots to compensate for the negative effects of grazing (18). In contrast, forbs generally invest more in shoots and leaves and create longer less-dense tap root systems. This together, leads to differences in the microbiomes grasses and forbs typically create around their roots (19–21). We further speculate that the microbiomes of forbs would be temporarily more stable due to their root morphology and exudation patters, yet we are not aware of any study showing this.

Plants have specific effects on the soil microbiome, and this microbiome in turn affects the growth of other plants growing in the same soil and their interactions with other organisms (20, 22, 23), Thus, the temporal variation in plant-associated microbiomes may have ecosystem-level consequences and affect the presence and coexistence of plants (24). Temporal dynamics of soil microbial communities may also vary between different types of microorganisms. Due to the relatively short generation times of bacteria, they are assumed to respond very rapidly to environmental changes, while especially hyphal growing fungi take longer to respond to changes (25–27). Many studies have also shown that in soils, fungi are more stable than bacteria during disturbances such as drought (28, 29). Recently, it was shown that soil fungi exhibit seasonal turnover, but it is unclear what mechanisms drive this and how long such turnover takes (30). We speculate that microbial communities will undergo succession in much shorter time intervals than, for example, plants due to relatively shorter generation times. Similar to succession in plants, in the long term, microbial communities are thought to develop toward a stable “equilibrium” composition (31). However, over relatively short time periods, changes in abiotic and biotic factors such as weather, season, and plant species can lead to gradual or more abrupt changes in microbial community composition due to variation in soil moisture and temperature or resource quality (32, 33).

Despite some key differences between plant and microbial succession, both are likely shaped by similar processes, such as dispersal limitation, drift, competition, and facilitation (34). Therefore, we used several concepts derived from plant ecology to test if they also apply to microbial succession. Specifically, we tested for nestedness and turnover (35) and appearance and disappearance, the microbial equivalent of colonization and extinction (36). Turnover indicates that species are replaced by others over time, while nestedness shows that the earlier community is a subset of the later community and vice versa (35). Our overarching goal was to better understand how soil microbial community composition changes over time in response to plant species and functional group identity. We tracked changes in soil fungal and bacterial communities in monocultures of three grass and three forb species at six time points during an entire year. We initiated the experiment with one common soil that was divided across 30 mesocosms (200 liters), each planted with monocultures of seedlings of one of the six plant species. The mesocosms were kept outdoors, which enabled us to follow both natural variation across seasons as well as variation in time. We collected soil samples 1, 2, 3, 6, 9, and 12 months after establishment of the experiment and analyzed the community composition and changes in similarity over time. The soil microbial communities were analyzed using ITS2 and 16S rRNA regions.

We analyzed two major types of soil microbes, bacteria and fungi, to examine if their community assembly is driven by similar factors. Our expectation was that fungi would be more stable over time due to their relatively longer generation times, while bacteria would be more dynamic since they respond more to environmental changes. As our study started with a relatively homogeneous soil (i.e., a common pool of microbes), we expected that at the beginning of the experiment, the selection by plants would be stochastic, as plants are small and rhizosphere areas are limited. Over time, plant species were expected to create species-specific microbiomes (deterministic) (37–39) and microbial composition would differ between grasses and forbs. We hypothesized that microbiomes under grasses would have a higher turnover rate due to the growth style of grasses. We expected time effects on the soil microbiome to be deterministic (e.g., through a seasonal pattern) and stochastic (e.g., by dispersal limitation or drift) (38, 40).

## RESULTS AND DISCUSSION

### Microbial diversity and abundance.

**FIG 1:**
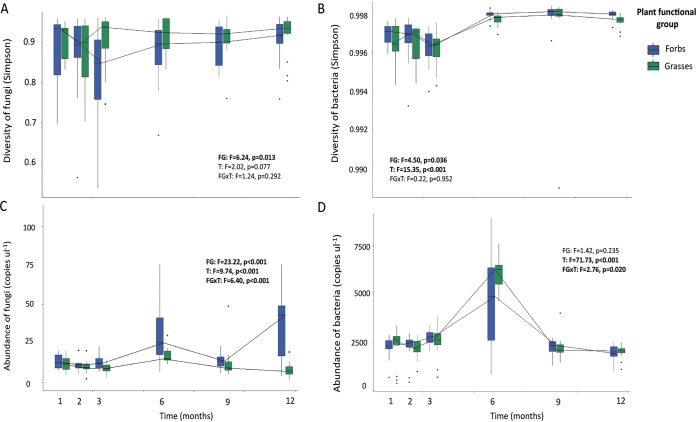
Alpha-diversity and abundance of fungi (A and C) and bacteria (B and D) in time (T) and between plant functional groups (FG) forbs (blue) and grasses (green). The Simpson diversity index was used to calculate the diversity of fungal phylotypes and bacterial OTUs, and copy numbers derived from quantitative PCR (qPCR) were used to estimate the abundance of the microbes. Tukey box-and-whisker plots show the medians (horizontal lines) and the quartiles (boxes) of data, and the whiskers show all variation. The lines through medians for both functional groups over time are also presented. The results from linear mixed models are given in each panel, and significant effects are marked in bold.

We first investigated the effects of time, plant species, and plant functional group (grasses versus forbs) on the diversity and abundance of soil bacteria and fungi. The diversity measured as Simpson inverse diversity of fungi was only marginally affected by time (*F* = 2.02, *P* = 0.077) (Fig. 1A), while Simpson inverse diversity of bacteria was greatly affected by time (*F* = 15.35, *P* < 0.001) (Fig. 1B). We expected that bacteria would be enriched due to the establishment of the plants and the increase in rhizosphere area over time. Bacterial diversity indeed increased over time and stabilized after 6 months. This is later than we expected based on the literature, as typically, time effects on bacterial diversity are investigated for only a few months (41, 42), potentially missing the time point with highest diversity.

Similarly as for diversity, we hypothesized that the microbial abundance would increase over time under favorable conditions. The highest abundance of bacteria was observed 6 months (November) after the start of the experiment, but thereafter, abundance of bacteria decreased again and returned to the initially recorded levels (Fig. 1D). For fungi, the highest abundance was also measured at 6 months, but abundance of fungi was also high 12 months after the start, in the soils in which forbs were growing (Fig. 1C). Abundance of bacteria generally increased faster in containers with grasses than in containers with forbs. The high abundance of fungi and bacteria at 6 months suggests that the microbial dynamics follow the seasonal dynamics of the plants (33, 43, 44). In November, just after the end of the growing season, air temperature was declining as winter approached (see Fig. S1 in the supplemental material). At this time, most plants had not yet gone dormant but had started senescing (see Fig. S2), resulting in ample litter material on the soil surface that can be processed by decomposer organisms. We hypothesized that the higher abundance of both bacteria and fungi observed in November was the result of both rhizosphere and detritusphere organisms that were both active at this stage due to the large amount of resources available (33), but further research over longer time periods (multiple years) is needed to separate the successional effects from seasonality.

10.1128/mBio.02635-19.1 FIG S1 Daily daytime temperature in Celsius 10 cm above soil for all mesocosms. Sampling moments are marked in the graph with arrows. Download FIG S1, TIF file, 0.6 MB. Copyright © 2019 Hannula et al. 2019 Hannula et al. This content is distributed under the terms of the Creative Commons Attribution 4.0 International license.

10.1128/mBio.02635-19.2 FIG S2 Plant community development over time. Pictures from all 30 mesocosms were taken on the same day, six times during the year. All replicate mesocosms (*n* = 5) are presented per block. The white sensors in the pictures are the temperature and moisture loggers. Download FIG S2, TIF file, 2.7 MB. Copyright © 2019 Hannula et al. 2019 Hannula et al. This content is distributed under the terms of the Creative Commons Attribution 4.0 International license.

Plant presence (the so-called “rhizosphere effect”) and plant diversity have been shown to affect both the diversity and abundance of soil microbes (39, 45). We expected that the different plant species would select their own microbiomes from the existing microbial pool in the soil (46, 47) and that we would detect plant species-specific effects on the diversity and abundance of the microorganisms. Diversity of both bacteria and fungi varied between plant species and functional groups (Fig. 1A and B and S3), but there were no interactive effects between time and plant species or plant functional group. Fungal diversity was consistently higher in grass than in forb soils, which is opposite to the abundance data (Fig. 1C). Plant functional group did not significantly affect the abundance of bacteria, but there was a weak interaction between time and plant functional group on bacteria. There was, however, a significant effect of plant species on the abundance of bacteria. Especially in Jacobaea vulgaris soil, the abundance of bacteria was lower 6 months after the start of the experiment, while the number of fungal copies was highest at this time point (Fig. S3). This is potentially due to the better ability of fungi than bacteria to defend against chemicals released by *Jacobaea vulgaris*, especially after prolonged plant growth (48, 49). Earlier studies reported no significant effects of plant species identity of monocultures on bacterial diversity (50). We concur with this finding for some of the time points measured but also show that at other time points, plant species differed in their effects on both diversity and abundance of bacteria.

10.1128/mBio.02635-19.3 FIG S3 Alpha-diversity and abundance of fungi (A and C) and bacteria (B and D) in time (T) and between plant species (S). Simpson diversity index was used to calculate the diversity of fungal phylotypes, and bacterial OTUs and copy numbers derived from qPCR were used to estimate the abundance of the microbes. Tukey box-and-whisker plots show medians and the quartiles of data, and the whiskers show full variation. The lines through medians in time are presented. The results from linear mixed models are given in the figure, and significant effects are marked in bold. Colors represent different plant species. Green colors represent grass monocultures and blue forb monocultures. AP, *Alopecurus pratensis*; HL, *Holcus lanatus*; FO, *Festuca ovina*; HR, *Hypochaeris radicata*; JV, *Jacobaea vulgaris*; TO, *Taraxacum officinale*. Download FIG S3, TIF file, 0.5 MB. Copyright © 2019 Hannula et al. 2019 Hannula et al. This content is distributed under the terms of the Creative Commons Attribution 4.0 International license.

### Plant species and functional group effects on soil microbiomes over time.

**FIG 2:**
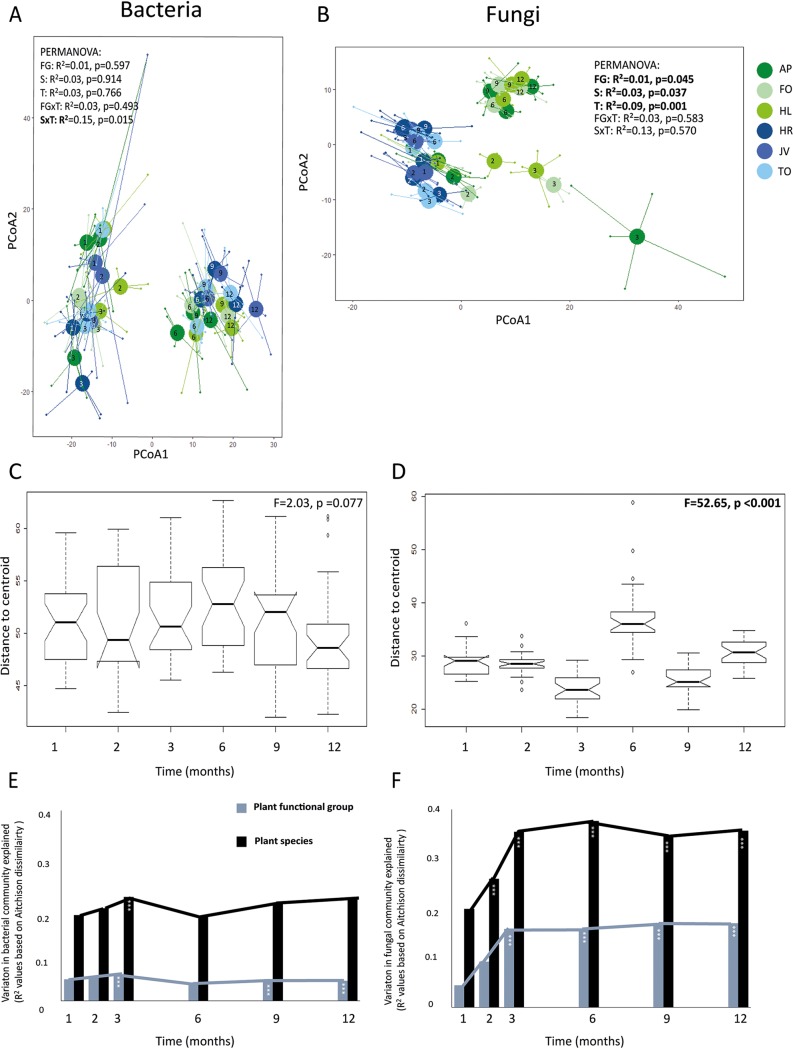
Bacterial (A) and fungal (B) community structures in time (T) and between plants species (S) and functional groups (FG) evaluated with log-centered ratio (Aitchison) analysis with principal-coordinate analysis (PCoA). Sampling times are marked within the centroids with numbers 1 to 12, and colors of the centroids and points represent plant species. Green colors are grass species and blue colors are forb species. Beta-dispersion of bacteria (C) and fungi (D) in time measured as distance to centroid between plant species each sampling time based on Aitchison distances. Beta-dispersion within plant species is shown in Fig. S4 in the supplemental material. The variation explained in bacterial (E) and fungal (F) communities per plant species (black bars) and plant functional groups (gray bars) in time estimated with PERMANOVA on Aitchison distances. ***, *P* < 0.001; AP, *Alopecurus pratensis*; HL, *Holcus lanatus*; FO, *Festuca ovina*; HR, *Hypochaeris radicata*; JV, *Jacobaea vulgaris*; TO, *Taraxacum officinale*.

Plants shape the composition of their rhizospheres in species-specific ways (41, 42, 45). We evaluated if these effects of plant species on soil fungi and bacteria are temporally and seasonally stable and at which time point these effects were the strongest. Using centered-log-ratio (CLR)-transformed data (51), we detected a significant interaction between sampling time and plant species identity on the community structure of bacteria (Fig. 2A). Bacterial communities were divided into two distinct types: one observed in months 1 to 3 and another one in months 6 to 12. The change that occurred between months 3 to 6 coincided with an increase in both bacterial diversity and abundance. For fungi, the community structure strongly responded to plant species identity, plant functional group, and time (Fig. 2B). At later sampling times (i.e., after 6 months), fungal community structure separated clearly between grass and forb monocultures. Beta-dispersion was used to examine if the dissimilarity between time points and plant species was driven by compositional differences or by differences in relative abundance of communities with similar composition (52). For fungi, the difference in beta-dispersion among time points was much larger than for bacteria, and this effect was significant (Fig. 2C and D). For both, the greatest divergence from the centroid occurred at 6 months. This confirms the finding that plant species had the greatest effect on both the fungal and bacterial communities in November after 6 months of plant growth. We speculate that soil microbial communities are relatively redundant until the peak in root and leaf senescence of the plants that grow in the soil, and that this then spurs the divergence in microbial communities.

10.1128/mBio.02635-19.4 FIG S4 Beta-dispersion of bacteria and fungi within time points divided per plant species. Download FIG S4, TIF file, 0.5 MB. Copyright © 2019 Hannula et al. 2019 Hannula et al. This content is distributed under the terms of the Creative Commons Attribution 4.0 International license.

The amount of variation explained by plant species and functional groups at each time point was determined using permutational analysis of variance (PERMANOVA) based on Aitchison distances. The variation in fungal communities explained by plant species and plant functional group increased from the first month (in June) to the third month (in August) and then remained relatively constant. Plant species explained most of the variation in fungal communities after 6 months (in November: *F* = 2.53, *R*^2^ = 0.38, *P* < 0.001), while plant functional group explained most of the variation after 9 months (in February: *F* = 5.54, *R*^2^ = 0.17, *P* < 0.001) (Fig. 2F). For bacteria, the amount of variation explained by plant species and plant functional group was much lower than for fungi, and plant functional group explained a significant part of the composition of the bacterial community only 3 months after the start (August: *F* = 1.51, *R*^2^ = 0.06, *P* < 0.001) (Fig. 2E). Plant species explained the bacterial community composition significantly at time points 3, 9, and 12 months (coinciding with August, February, and May, respectively) (Fig. 2). The results clearly show that the effects of plants on the soil community increased over time, at least initially. This can potentially explain the discrepancy of results obtained in different plant-soil feedback studies (53), and we recommend strongly to consider seasonality and duration of conditioning phase as main factors affecting the outcome of plant-soil microbiome studies. Furthermore, for fungi, the effect of time seems to be cumulative: it took 2 to 3 months for the plants to shape a specific mycobiome in the soil, and thereafter, these effects increased over time. In our study that took 12 months, we cannot distinguish between the effects of season and time of conditioning. However, the fact that the soil communities and, especially, the fungal community after 12 months of plant growth had not returned to the original state but instead shifted in plant species-specific directions over time suggests that presence of the plants and the duration of plant growth are the main drivers of soil microbial community composition.

**FIG 3:**
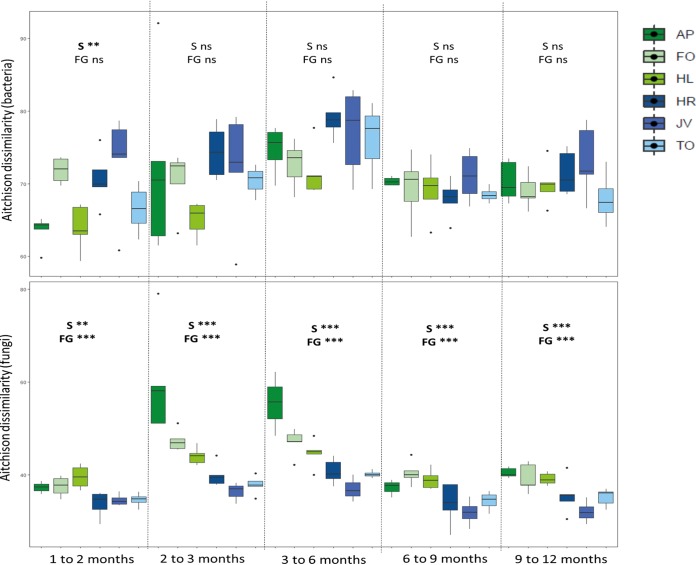
Aitchison dissimilarities of bacterial and fungal communities between time points per plant species. Significance of plant species (S) and plant functional group (FG) derived from linear mixed models is presented. **, *P* < 0.005; ***, *P* < 0.001. The grass monocultures are shown in green colors and forb monocultures in blue colors. Tukey box-and-whisker plots show the medians (horizontal lines) and the quartiles (boxes) of data, and the whiskers show all variation. AP, *Alopecurus pratensis*; HL, *Holcus lanatus*; FO, *Festuca ovina*; HR, *Hypochaeris radicata*; JV, *Jacobaea vulgaris*; TO, *Taraxacum officinale*.

To unravel the effects of plant species and plant functional group in driving the observed dissimilarities of soil microbial communities between time points, we calculated the rates of change in fungal and bacterial community dissimilarities between consecutive time points (Fig. 3). For fungi, differences were larger for grasses than for forbs, and grasses changed more between time points than forbs. The highest dissimilarity was observed for the grass Alopecurus pratensis, which indicates that this species had the strongest effect on the community structure of fungi in the soil. For bacteria, a plant species effect was detected in distances between the 1- and 2-month samplings (*F* = 5.12, *P* = 0.002): Festuca ovina, *J. vulgaris*, and Hypochaeris radicata had larger effects on community development than *A. pratensis*, Holcus lanatus, and Taraxacum officinale. For both bacteria and fungi, distances were significantly affected by time (*F* = 8.26 and *F* = 14.93, *P* < 0.001, for bacteria and fungi, respectively) and were highest between the 3- and 6-month measurements. Previous work has shown that plants that belong to different plant functional groups generally create divergent soil legacy effects on future plant growth (21, 54, 55) and that microbial shifts are more pronounced in soil fungi than in bacteria (21). Our results suggest that the effects of plant growth on the community structure of fungi remain fairly consistent over time. Future studies should examine if such temporal changes in fungal community structure result in differences in soil functions and translate into altered plant community dynamics.

**FIG 4:**
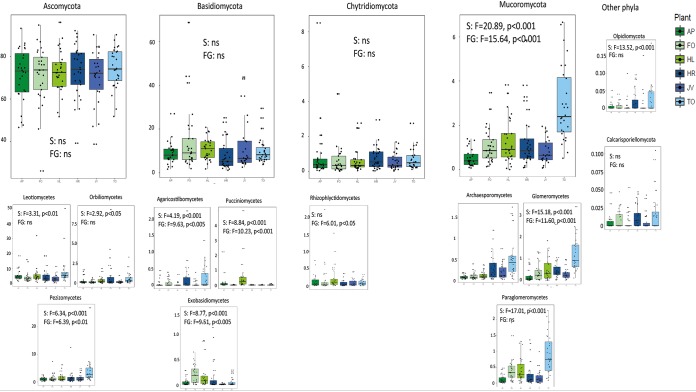
Relative abundances of fungal phyla (top) and classes (bottom) significantly affected by plant species or plant functional group across time points. Statistical significance of the effects of plant species (S) and plant functional group (FG) derived from a generalized linear mixed model (GLMM) is also presented in the figure. Grass monocultures are presented in green colors and forb monocultures in blue colors. Tukey box-and-whisker plots show the medians (horizontal lines) and the quartiles (boxes) of data, and the whiskers show all variation. AP, *Alopecurus pratensis*; HL, *Holcus lanatus*; FO, *Festuca ovina*; HR, *Hypochaeris radicata*; JV, *Jacobaea vulgaris*; TO, *Taraxacum officinale*.

We subsequently analyzed the groups of bacteria and fungi that were affected by plant species identity and plant functional group using linear mixed-effects models with arcsine-square-root-transformed relative abundance values. Plant functional group had a significant effect on the relative abundances of 11 bacterial phyla (of 20) and two fungal phyla (of eight) (Fig. 4; see also Table S1). For fungi, 11 classes were significantly affected by plant functional group (Fig. 4). We detected no significant interactions between time and functional group for bacteria, while for fungi, the interaction between time and plant species was a significant factor explaining the relative abundances of Mucoromycota and Basidiomycota and multiple classes (Table S1). Plant species identity had an effect on relative abundances of 16 bacterial phyla and 11 fungal classes. These taxa were generally the same as for plant functional groups.

10.1128/mBio.02635-19.8 TABLE S1 Bacterial phyla influenced by plant species, plant functional group (grasses versus forbs), time, and their interactions. Download Table S1, DOCX file, 0.1 MB. Copyright © 2019 Hannula et al. 2019 Hannula et al. This content is distributed under the terms of the Creative Commons Attribution 4.0 International license.

The most notable group of fungi responding strongly to plant species identity and plant functional group was members of the subphylum Glomeromycotina. Especially, *T. officinale* enriched the soils with Paraglomeromycetes and Glomeromycetes, while Archeosporomycetes were generally found more in soils in which forbs were grown. Plant species-specific selection of Glomeromycotina from the soil microbiome was shown earlier (e.g., see references 56 and 57). This can potentially influence plant-soil feedbacks due to differences in nutrient acquisition strategies between plants (58) and influence the composition of plant communities. We further investigated at the level of individual bacterial operational taxonomic units (OTUs) and fungal phylotypes if specific species of microbes were selected by certain plant species or plant functional groups. Only OTUs or phylotypes present in at least 3 of the 5 replicate mesocosms per monoculture and present in at least two of the time points were included. Approximately 30% of microbes were consistently present in soils of all plant species forming a stable “core microbiome” in these soils (see Fig. S5). Less than 2% of bacterial OTUs and approximately 5% of fungal phylotypes that were found during at least two time points were specific to plant species, indicating that observed differences in the community structure can be mostly explained by changes in the relative abundances of taxa and not by recruitment of new species by plants. The proportion of OTUs and phylotypes that were unique to plant functional group was even lower, less than 1% of the OTUs and 4% of the phylotypes. Forbs had more unique forb-specific bacterial OTUs than grasses, while grasses had more unique fungal phylotypes than forbs (Fig. S5). Furthermore, *J. vulgaris* monocultures had the most unique OTUs, and *H. lanatus* monocultures had the most unique phylotypes, indicating they most strongly select and create unique microbiomes. Grass-specific fungal phylotypes mainly belonged to the ascomycetes and included several known grass-pathogens (such as *Didymella graminicola*, Myrmecridium phragmitis, and Tilletiaria anomala) which can potentially cause negative feedback with subsequent grass species grown in the soils. Forb-specific bacteria were mainly *Bacteroidetes* and *Alphaproteobacteria*, which are dominant soil bacterial taxa (59).

10.1128/mBio.02635-19.5 FIG S5 Individual OTUs and phylotypes shared between plant species and plant functional groups. Download FIG S5, TIF file, 0.5 MB. Copyright © 2019 Hannula et al. 2019 Hannula et al. This content is distributed under the terms of the Creative Commons Attribution 4.0 International license.

### Succession of bacterial and fungal communities.

The coefficient of variation (CV) values representing temporal variability in bacterial and fungal diversity were not significantly affected by plant species (*F* = 3.01, *P* = 0.10 and *F* = 0.18, *P* = 0.67, respectively) (see Fig. S6) or plant functional group (*F* = 1.78, *P* = 0.17 and *F* = 0.78, *P* = 0.55, respectively) indicating that microbial diversity is not *per se* affected by time in a plant species-specific way even when the community structure overall is affected.

10.1128/mBio.02635-19.6 FIG S6 Temporal variability of diversity of bacteria (A) and fungi (B) represented as CV values for each plant species (S). Colors indicate plants species: green colors represent grasses and blue colors forbs (plant functional groups [FG]). Tukey box-and-whisker plots show the medians (horizontal lines) and the quartiles (boxes) of data, and the whiskers show all variation. AP, *Alopecurus pratensis*; HL, *Holcus lanatus*; FO, *Festuca ovina*; HR, *Hypochaeris radicata*; JV, *Jacobaea vulgaris*; TO, *Taraxacum officinale*. Download FIG S6, TIF file, 0.4 MB. Copyright © 2019 Hannula et al. 2019 Hannula et al. This content is distributed under the terms of the Creative Commons Attribution 4.0 International license.

**FIG 5:**
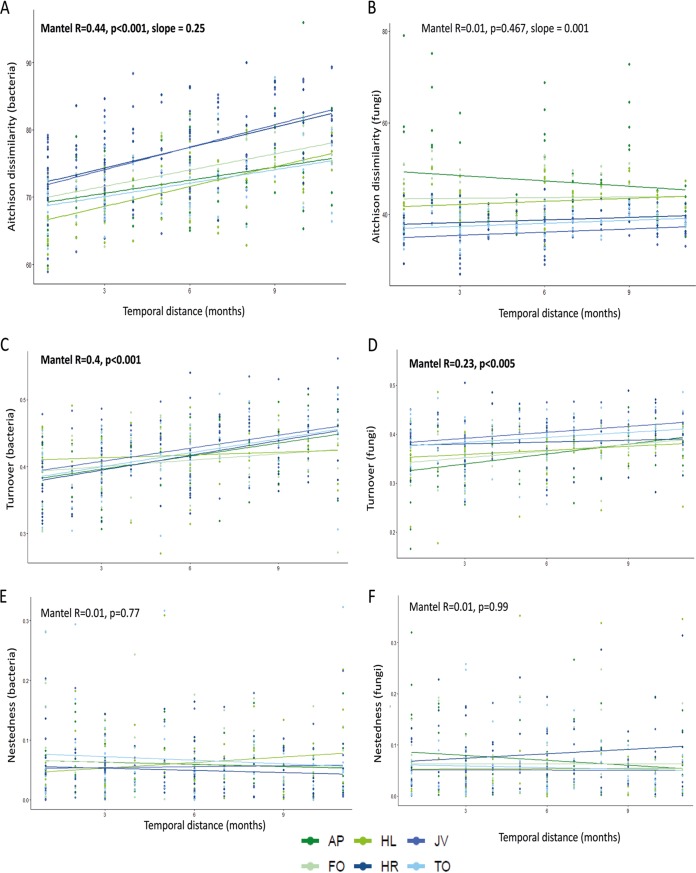
Mantel distances of bacterial (A) and fungal (B) dissimilarity with temporal distance and Aitchison dissimilarities. The temporal variation is further divided into turnover (C and D) and nestedness (E and F) for bacteria (C and E) and fungi (D and F) in time. The compositional variance of the bacterial and fungal communities calculated using Sorenson dissimilarity was portioned with Simpson pairwise dissimilarity to calculate the turnover and nestedness of the community (35), and a Mantel test was performed to explore the correlation between temporal distance and turnover or nestedness. Dissimilarities of communities over time, turnover, and nestedness were calculated separately for all plant species, and they are visualized with different colors in the figure. Green represents grass monocultures and blue forb monocultures. AP, *Alopecurus pratensis*; HL, *Holcus lanatus*; FO, *Festuca ovina*; HR, *Hypochaeris radicata*; JV, *Jacobaea vulgaris*; TO, *Taraxacum officinale*.

Recent studies showed that both the overall fungal (30) and Glomeromycotina communities in soils show successional patterns (60) at scales of months and years. Furthermore, using chronosequence approaches, both fungi and bacteria have been shown to respond to plant succession (22, 34, 61). Here, we did not detect a significant increase in community dissimilarity over time for total fungi, which indicates that our communities were not undergoing succession (Fig. 5B; see also Fig. S7). This discrepancy in results is likely due to differences in spatial and temporal scales as well as the set-up of the systems. Here, we restricted our study to mesocosms with one soil type and six plant monocultures, and it is plausible that the temporal variation is larger when more complex systems such as natural forests or grasslands are sampled (22, 30, 34). In contrast to fungi, bacterial communities grew more dissimilar with increasing distance in time of sampling (Mantel test: *R* = 0.6, *P* < 0.001, slope = 0.035; and *R* = 0.44, *P* < 0.001, slope = 0.025) (Fig. 5A). Moreover, forbs affected the turnover of soil bacterial communities more than grasses (slope of 0.040 versus slope of 0.032) (Fig. 5A). The highest rate of change of bacterial communities was found in the soils in which the forb *J. vulgaris* was grown.

10.1128/mBio.02635-19.7 FIG S7 Time decay curves for bacteria (A) and fungi (B). Colors represent different plant species. Green colors represent grass monocultures and blue indicates forb monocultures. AP, *Alopecurus pratensis*; HL, *Holcus lanatus*; FO, *Festuca ovina*; HR, *Hypochaeris radicata*; JV, *Jacobaea vulgaris*; TO, *Taraxacum officinale*. Download FIG S7, TIF file, 0.4 MB. Copyright © 2019 Hannula et al. 2019 Hannula et al. This content is distributed under the terms of the Creative Commons Attribution 4.0 International license.

We subsequently separated differences in bacterial communities over time to turnover and nestedness. Mantel tests comparing turnover and time showed that for both bacteria and fungi, indeed, turnover plays an important role in shaping the community (Fig. 5C and D), but turnover is stronger for bacteria and is plant species specific. For *H. lanatus* (Mantel: *R* = 0.1, *P* = 0.288) and *F. ovina* (Mantel: *R* = 0.22, *P* = 0.054), turnover did not play a significant role in shaping their bacterial communities, but for the other four plant species, turnover significantly affected the bacterial community. In general, slopes in turnover were steeper for forb species than for grass species (slopes of *R* = 0.43 compared to *R* = 0.24 for grasses); although, for both groups of plant species, turnover played a significant role. Even though fungi were not affected by time, we tested if they were influenced by turnover or nestedness. For fungi, the communities in soils from *F. ovina* (Mantel: *R* = 0.32, *P* < 0.01), *J. vulgaris* (Mantel: *R* = 0.34, *P* < 0.01), and *A. pratensis* (Mantel: *R* = 0.40, *P* < 0.005) were significantly structured by turnover, while the fungal communities in the soils under the three other monocultures were not affected by turnover. For fungi, turnover was weaker in general. Contrary to what was observed for bacterial community turnover, for fungi, grasses had a generally steeper slope (*R* = 0.29, *P* < 0.001) than forbs (*R* = 0.18, *P* < 0.05), indicating that community structuring of fungi in time is plant species specific. Nestedness did not play a significant role in shaping bacterial or fungal communities in time (Fig. 5E and F). Earlier studies have identified both turnover and nestedness as forces driving Glomeromycotina and bacterial communities (60, 62). Here, we show that both bacterial and fungal communities are driven by turnover but that this is specific to plant species and that its strength varies among plant functional groups.

### Immigration and extinction.

Lastly, we investigated the immigration and extinction patterns within the microbial communities. We looked at this at two different levels. First, we examined which fungal classes and which bacterial phyla were affected by time and plant species and then examined individual “species” of microorganisms that were present at the beginning versus at the end of the experiment.

**FIG 6:**
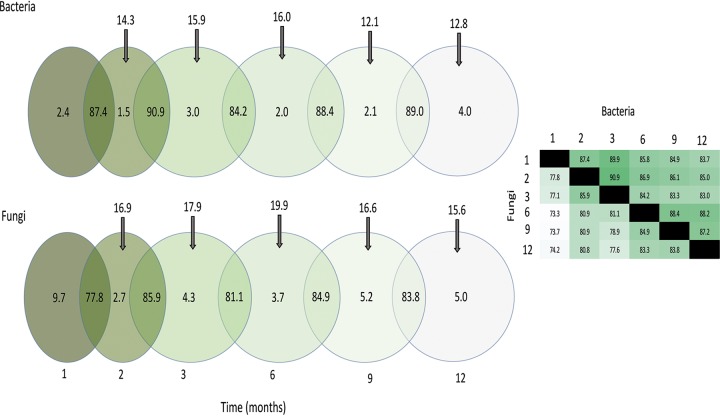
Immigration and extinction. The percentages of shared and unique bacterial OTUs and fungal phylotypes between time points, unique to time points, and new in each time point (indicated with arrows). Most of the OTUs and phylotypes are shared between two consecutive time points. The percentage of OTUs shared between all time points is shown on the right.

We detected 48.1% of bacteria and 38.5% of fungi at all sampling points. Approximately 84% to 91% of bacterial OTUs and 78% to 86% of fungal OTUs were shared between consecutive sampling times, making the loss in the community (extinction rate) between 9% and 22%. The largest extinction rate was detected for fungi between 1 and 2 months and, for bacteria, between 3 and 6 months. The immigration rates, measured as recruitment of new species that were not present at the previous time point, were between 12% and 16% for bacteria and between 15% and 20% for fungi. For both groups of organisms, the highest immigration rate was between 3 and 6 months, while the lowest rate for fungi was between 9 and 12 months and, for bacteria, between 6 and 9 months (Fig. 6). Furthermore, very few OTUs and phylotypes were found only at one time point (between 1.5% and 9.7% for fungi), indicating that the immigrated microbes established well. On average, any two sampling points shared 86.3% of bacteria and 79.6% of fungi. For bacteria OTUs, the largest difference was detected between August and May (83.0% OTUs shared), while the smallest difference was between July and August (90.9% OTUs shared). In general, the closer two sampling points were in time, the higher the number of shared OTUs. This low dispersal/immigration rate (<20%) and large proportion of OTUs shared between sampling times indicate that the observed patterns in time are most likely deterministic, as often assumed for microbial communities (37, 38).

**FIG 7:**
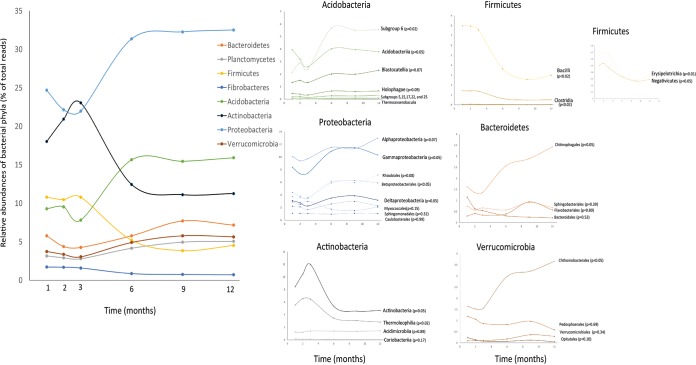
Bacterial phyla, and orders within these phyla, that were significantly affected by time of sampling evaluated using Fisher’s G.

We used Fisher’s G to estimate which phyla, classes, and orders were significantly affected by time across plant species. For fungi, none of the taxa were significantly affected by time. For bacteria, on the other hand, clear seasonal dynamics were observed. At the level of phyla, eight phyla were significantly affected by temporal dynamics (Fig. 7). Especially evident was a shift in communities dominated by *Actinobacteria*, *Chloroflexi*, and *Firmicutes* at 1 to 3 months to communities with *Acidobacteria*, *Proteobacteria*, and *Bacteroidetes* at 6 to 12 months (Fig. 7). Furthermore, within all these phyla, we identified the main classes and orders causing the observed patterns (Fig. 7). The increase in abundance of *Acidobacteria* at 6 months (November) was related to an increase in abundance of bacteria from the *Acidobacteria* subgroup 6 and the *Acidobacteriia*. The decrease in *Actinobacteria* was mainly explained by a decrease in *Thermoleophilia* and the class *Actinobacteria*. The pattern observed for *Proteobacteria* was more mixed: *Alphaproteobacteria*, *Gammaproteobacteria*, and Deltaproteobacteria showed similar patterns for the first 9 months, but between 9 and 12 months, the proportion of *Alphaproteobacteria* continued to increase, while the proportions of *Gammaproteobacteria* and Deltaproteobacteria started to decrease. For *Firmicutes*, all classes were showing significant temporal patterns, and all were decreasing over time.

### Conclusions.

Fungal community structure was most affected by plant species and functional group identity, while the bacterial community changed drastically between time points and, especially, between 3 and 6 months. Moreover, for bacteria, time was an important factor in shaping the community structure. Importantly, this shift in bacterial communities from one state to another (63) is unlikely to change the functioning of the soils, as we did not detect large numbers of species disappearing (becoming extinct) in the system nor did we detect a large wave of species immigrating; rather, we observed changes in their relative abundances (64). Plant species differed in their selective pressure on soil fungi over time, and grasses had a stronger effect on the fungal communities than forbs. Our results emphasize the importance of measuring microbial diversity over longer time periods and the importance of repeated sampling in characterization of soil microbial communities. Temporal changes in microbial populations could interact with the plant community to affect vegetation composition, leading to knock-on effects for ecosystem processes such as decomposition or carbon sequestration. The next generation of experiments should focus on tracking soil microbial community development in mixed plant communities over time and over longer periods of time to separate the effects of succession from the effects of seasonality.

## MATERIALS AND METHODS

### Sampling and sequencing.

Thirty containers (48 cm by 80 cm by 50 cm) were filled with soil that was sieved through a 32-mm sieve. The soil used in the experiment is characterized as holtpodzol sandy loam (84% sand, 11% silt, 2% clay, ∼3% organic matter, pH 5.9, 1,150 mg kg^−1^ N, 61 mg P_2_O_5_ 100 g^−1^, 2.4 mmol K kg^−1^) collected from a grassland near Lange Dreef, Driebergen, The Netherlands (52°02′N, 5°16′E). As a living inoculum from a species-rich well-developed grassland, we added 20 kg soil from a natural grassland (“De Mossel,” Ede, The Netherlands, 52°04′N, 5°45′E) on the top of each container. This soil was sieved through a 10-mm sieve and characterized as holtpodzol sandy loam (94% sand, 4% silt, 2% clay, ∼5% organic matter, pH, 5.2, 1,060 mg kg^−1^ N, 75 mg P_2_O_5_ 100 g^−1^ P, 1.9 mmol K kg^−1^).

On 1 May 2017, monocultures of six plant species were planted as seedlings to the soils. A total of 100 seedlings were planted per monoculture in each container. Each species was planted in 5 replicate blocks in a randomized block design. We used three grass species (Holcus lanatus, *Festuca ovina*, *Alopecurus pratensis*) and three forb species (*Hypochaeris radicata*, *Jacobaea vulgaris*, and *Taraxacum officinale*) all naturally occurring in the location where the soil was sampled.

The experiment was conducted in the common garden at the Netherlands Institute of Ecology (NIOO-KNAW, Wageningen, The Netherlands; 51°59′N, 5°40′E). Mesocosms were watered regularly during the summer months to avoid drying out. The daily daytime temperature at the location is shown in Fig. S1 in the supplemental material. Plant development in the monocultures was followed monthly by taking photos (Fig. S2). Over the course of 1 year (June 2017 to June 2018), six soil samples were taken from each container at regular intervals (12 cm deep, 7 mm diameter), pooled and homogenized per time point, and immediately stored at −20°C until molecular analysis. Samples were collected first monthly (June, July, and August) and then every 3 months (November, February, and May), always during the first week of the month.

DNA was extracted from 0.75 g of soil using the PowerSoil DNA Isolation kit (Qiagen, Hilden, Germany) according to the manufacturer’s protocol. Fungal and bacterial DNA was amplified and sequenced using MiSeq PE250 (65–68). For details, see supplemental materials and methods.

### Quantification of bacteria and fungi.

We quantified the copy numbers of bacterial and fungal marker genes in the same DNA samples as used for sequencing. Quantitative PCR assays were performed using the Rotor-Gene SYBR green PCR kit (Qiagen, Hilden, Germany) and the primers ITS4ngs and ITS3mix for fungi (68) and the primers eub338 and eub518 for bacteria (69). For details, see supplemental materials and methods.

### Bioinformatic and statistical analyses.

Bacterial and fungal sequences were analyzed using the PIPITS pipeline and the Hydra pipeline, respectively (70, 71). For details on the pipelines, see supplemental materials and methods.

It was recently pointed out that microbiome data from high-throughput sequencing (HTS) is compositional and should not be treated as counts (72). Therefore, we only used compositional data in our analyses (51). We converted both bacterial and fungal data with centered log ratio (CLR) using CoDaSeq (51). Community similarity was based on both Aitchison distances, and the effects of plant species identity and time on the fungal and bacterial communities were analyzed using permutational analysis of variance (PERMANOVA) using the vegan package (73). To test the homogeneity of the communities over time (74), beta-dispersion was calculated using the betadisper function in the vegan package in R (73). To visualize the effects of time and plant species on bacterial and fungal communities, a principal-coordinate analysis (PCoA) was performed using the vegan package.

Fungal and bacterial diversity was estimated using the inverse Simpson index. To compare temporal variability in the microbial diversity with potentially different diversities in the different monocultures, we calculated the coefficient of variation (CV) of within-container diversity for each plant monoculture across time points. In using the coefficient of variation, we compared variability in diversity rather than absolute measures of diversity in time, which provides an indication of the plant species effects across time points. This was performed to examine if a plant species or plant functional group would exert a larger effect on microbial diversity over time than other plant species or the other functional group. The sample size was the same for all samples, and individual containers were used analyzed separately.

We further explored the temporal dynamics in diversity by plotting the fungal and bacterial richness over time. The relationships between time and the abundance of initially dominant and initially rare taxa and OTUs were explored using linear mixed-effects models, which included random effects for monoculture identity using the lme4 package (75). For the effects of plant species, container number was used as a random factor, and for the effects of plant functional group, plant species was nested in block. To obtain normality, the arcsine square root transformation was used for the relative abundance data of each taxa (76).

As analytical methods to evaluate partitioning of nestedness and turnover of beta diversity do not work with compositional data, for these analyses, filtered presence-absence data and Sorenson dissimilarity were used to calculate these variables (35) using the beta.pair command in the betapart package (77).

To investigate if the soil microbial community dissimilarity changed over time, we performed a time-decay curve analysis for each plant species (11) using an approach previously used in aquatic community studies (78). Specifically, a log-linear model was fitted between the change in community structure (assessed by pairwise Aitchison distances) and the number of months elapsed. Community dissimilarities were converted to similarities by subtracting from 100 and were subsequently log transformed. The slope of the log-linear model is the rate of community change, referred to as turnover (79). We also examined changes between two consecutive time points (from 1 to 2 months, from 2 to 3 months, etc.) by calculating the rate of change between two time points. This was performed by dividing the dissimilarity index between the two time points by the time between the observations.

A Mantel test was performed to explore correlations between changes in time and dissimilarities in Aitchison distances, turnover, and nestedness for both bacteria and fungi. We restricted the distances to distance within each container and examined both overall effects and plant species-specific effects. We used Pearson’s correlation coefficients with 999 permutations and used a Monte Carlo test to correct for random significance.

The R package GeneCycle was used to carry out a Fisher G test to determine the significance (*P* < 0.05) of the periodic components on the level of phyla, classes, and orders (80). For orders making up the majority of a class, no separate analysis was performed at order level (i.e., Orbiliales in Orbiliomycetes, Pezizales in Pezizomycetes, and Saccharomycetales in Saccharomycetes). *P* values were adjusted using Benjamini-Hochberg corrections for multiple testing (81), and phyla/classes/orders with an adjusted *P* value (false discovery rate [FDR]) of <0.05 were considered to be significantly affected by seasonality.

### Data availability.

Sequences created in this study were deposited in ENA with accession number PRJEB33273. The R codes used are available upon request.

10.1128/mBio.02635-19.9 DATA SET S1 OTU tables of bacteria and fungi as supplemental material. The raw data is deposited in ENA. Download Data Set S1, XLSX file, 9.7 MB. Copyright © 2019 Hannula et al. 2019 Hannula et al. This content is distributed under the terms of the Creative Commons Attribution 4.0 International license.

10.1128/mBio.02635-19.9 TEXT S1 Supplementary material and methods. Download TEXT S1, DOCX file, 0.1 MB. Copyright © 2019 Hannula et al. 2019 Hannula et al. This content is distributed under the terms of the Creative Commons Attribution 4.0 International license.
