# Supplementary material for: Time after Time: Temporal Variation in the Effects of Grass and Forb Species on Soil Bacterial and Fungal Communities
Source: mBio. 2019 Dec 17;10(6):e02635-19. doi: 10.1128/mBio.02635-19 (PMC6918080; doi:10.1128/mBio.02635-19)
Supplement: TABLE S1 [file mBio.02635-19-st001.docx]

**Supplementary table 1.** Bacterial phyla influenced by plant species, plant functional group (grasses vs. forbs), time and their interactions.
