# Supplementary material for: Time after Time: Temporal Variation in the Effects of Grass and Forb Species on Soil Bacterial and Fungal Communities
Source: mBio. 2019 Dec 17;10(6):e02635-19. doi: 10.1128/mBio.02635-19 (PMC6918080; doi:10.1128/mBio.02635-19)
Supplement: TEXT S1 [file mBio.02635-19-s0001.docx]

Supplementary materials and methods for manuscript:

**Time after time: Temporal variation in the effects of grass and forb species on soil bacterial and fungal communities**

Authors: S. Emilia Hannula, Anna M. Kielak, Katja Steinauer, Martine Huberty, Renske Jongen, Jonathan R. De Long, Robin Heinen & T. Martijn Bezemer

*DNA work and sequencing*

DNA was extracted from 0.75 g of soil using the PowerSoil DNA Isolation Kit (Qiagen, Hilden, Germany) following the manufacturer’s protocol, amplified for fungi and bacteria and sequenced using MiSeq PE250. For details see supplementary material and methods. The DNA quantity was measured using a Nanodrop spectrophotometer (Thermo Scientific, Hudson, NH, USA). Approximately 100 ng of DNA was used for a PCR. The primers ITS4ngs and ITS3mix targeting the ITS2 region of fungi (1) and the primers 515FB and 806RB (2-4) targeting the V4 region of the 16S rRNA gene in bacteria were used. Presence of PCR product was checked using agarose gel electrophoresis. The PCR products were purified using Agencourt AMPure XP magnetic beads (Beckman Coulter, Brea, CA, USA). Adapters and barcodes were added to samples using Nextera XT DNA library preparation kit set A & B (Illumina, San Diego, CA, USA). The final PCR product was purified again with AMPure beads, checked using agarose gel electrophoresis and quantified using a Nanodrop spectrophotometer before equimolar pooling. We pooled all fungal samples (180) in one sequencing run and divided the bacterial samples in two separate runs (90 samples each). A mock community, containing 10 fungal species, was included in each run to investigate the accuracy of the bioinformatics analysis. Libraries were sequenced using Illumina MIseq PE 250 at McGill University and Genome Quebec Innovation Center. Extraction negatives were used and further sequenced.

*qPCR*

T4 Gene 32 protein (Roche, Basel, Switzerland) was used to enhance the reaction and ensure similar amplification from all soils. The reaction mixtures were pipeted using a robot (Gorbett Research, Sydney, Australia) in 20 µl volume and contained 0.3 µM of each primer, 0.25 µl T4 and 1.0-10.0 ng template DNA. The cycling conditions for fungi were: 40 sec at 95 °C, 1 min at 55 °C and 1 min at 72 °C and for bacteria 40 sec at 95 °C, 1 min at 53 °C and 1 min at 72 °C. The samples were analysed on a Rotor-Gene 3000 machine (Gorbett Research, Sydney, Australia). Plasmids extracted from a pure fungal culture (*Hypholoma vesicular*) and a pure bacterial culture (*Collimonas fungivorans*), were serial diluted and used as a reference to calculate the copy numbers.

*Bioinformatic analysis*

In short, fungal sequences were paired using VSEARCH and quality was filtered using standard parameters. The ITS2 region was extracted using ITSx (5). Short reads were removed, and sequences were clustered based on a 97% similarity threshold using VSEARCH and chimeric sequences were removed by comparing with UNITE uchime database. The representative sequences were identified using the RDP classifier against the UNITE database (6, 7). For bacterial sequences, the VSEARCH was used to pair sequences, and cluster them. For classification, SINA classification was used with the SILVA database. Fungi were assigned to potential functions using FunGuild (8) and curated using inhouse databases (9). We define here that OTUs of bacteria based on 97% clustering threshold are units, while for fungi we use ‘phylotypes’ as surrogates for species. We used OTUs and phylotypes instead of exact sequence variants (ESVs), as we are not interested here in strain level variation in community structure and because on an ecologically relevant level, both show similar patterns (10). Prior to statistical analysis, we filtered out all sequences originating from organisms other than fungi and bacteria, including chloroplast and mitochondria. We removed OTUs that were present in less than five samples and had a relative abundance of less than 0.01% and samples that had less or more than five times the average read number.

References to supplementary material and methods:

1. Tedersoo L, Anslan S, Bahram M, Põlme S, Riit T, Liiv I, et al. Shotgun metagenomes and multiple primer pair-barcode combinations of amplicons reveal biases in metabarcoding analyses of fungi. MycoKeys. 2015;10.

2. Apprill A, McNally S, Parsons R, Weber L. Minor revision to V4 region SSU rRNA 806R gene primer greatly increases detection of SAR11 bacterioplankton. Aquat Microb Ecol. 2015;75(2):129-37.

3. Caporaso JG, Lauber CL, Walters WA, Berg-Lyons D, Huntley J, Fierer N, et al. Ultra-high-throughput microbial community analysis on the Illumina HiSeq and MiSeq platforms. The ISME journal. 2012;6:1621.

4. Parada AE, Needham DM, Fuhrman JA. Every base matters: assessing small subunit rRNA primers for marine microbiomes with mock communities, time series and global field samples. Environ Microbiol. 2016;18(5):1403-14.

5. Bengtsson-Palme J, Ryberg M, Hartmann M, Branco S, Wang Z, Godhe A, et al. Improved software detection and extraction of ITS1 and ITS2 from ribosomal ITS sequences of fungi and other eukaryotes for analysis of environmental sequencing data. Methods in Ecology and Evolution. 2013;4(10):914-9.

6. Koljalg U, Nilsson RH, Abarenkov K, Tedersoo L, Taylor AF, Bahram M, et al. Towards a unified paradigm for sequence-based identification of fungi. Mol Ecol. 2013;22(21):5271-7.

7. Abarenkov K, Henrik Nilsson R, Larsson KH, Alexander IJ, Eberhardt U, Erland S, et al. The UNITE database for molecular identification of fungi--recent updates and future perspectives. New Phytol. 2010;186(2):281-5.

8. Nguyen NH, Song Z, Bates ST, Branco S, Tedersoo L, Menke J, et al. FUNGuild: An open annotation tool for parsing fungal community datasets by ecological guild. Fungal Ecology. 2016;20(Supplement C):241-8.

9. Hannula SE, Morrien E, de Hollander M, Van der Putten WH, de Boer W, Van Veen JA. Shifts in rhizosphere fungal community during secondary succession following abandonment from agriculture. ISME J. 2017;11:2294-304.

10. Glassman SI, Martiny JBH. Broadscale Ecological Patterns Are Robust to Use of Exact Sequence Variants versus Operational Taxonomic Units. mSphere. 2018;3(4):e00148-18.
